# Effect of polyvinylpyrrolidone on sperm function and early embryonic development following intracytoplasmic sperm injection in human assisted reproduction

**DOI:** 10.1007/s12522-012-0126-9

**Published:** 2012-04-10

**Authors:** Yoku Kato, Yoshikazu Nagao

**Affiliations:** ^1^ Department of Animal Production Science, United Graduate School of Agricultural Science Tokyo University of Agriculture and Technology 183‐8509 Fuchu Japan; ^2^ University Farm, Faculty of Agriculture Utsunomiya University Shimokomoriya 443 321‐4415 Mohka Tochigi Japan

**Keywords:** Sperm capacitation, Embryo development, Polyvinylpyrrolidone (PVP), Clinical utility in vitro embryo production in human

## Abstract

The objective here was to review the effects of polyvinylpyrrolidone (PVP) upon sperm function and embryonic development in humans. PVP has been used successfully in intracytoplasmic sperm injection (ICSI) to facilitate the handling and immobilization of sperm for both domestic animals and humans. In our previous reports, PVP solution exists locally in embryos injected during the early developmental period, and also exerts influence over the developmental capacity of such embryos. In other reports, PVP causes significant damage to sperm membranes that can be detected by transmission electron microscopy, and has been associated with chromosomal abnormalities in pregnancy derived from ICSI embryos. In some Japanese clinics, PVP‐free media has been used for sperm immobilization in order to optimise safety. Consequently, it is strongly suggested that the success rate of fertilization and clinical pregnancy could be improved by using PVP‐free solution for human ICSI. In conclusion, our interpretation of the available data is to perform ICSI without PVP or select a lower concentration of PVP solution in order to reduce safety for pregnancy and children born via ICSI.

## Introduction

Polyvinylpyrrolidone (PVP) is a soluble polymer in water and made from *N*‐vinylpyrrolidone [1]. In the 1930s, the PVP patent was filed as one of the most attractive chemicals of acetylene chemistry [1]. PVP was first used for a blood plasma substitute and subsequently in a variety of applications in the fields of medicine, pharmacy, cosmetics and industry [1]. Povidone iodine is equally effective and could be preferred due to easy availability and lower cost for the objectives of those fields [2]. On the other hand, we experienced many documented cases of allergic reactions to PVP/povidone, especially for subcutaneous utility and when PVP has been touching autologous serum and mucous membranes [3, 4]. In another case, an allergic reaction to PVP was found in some people [5, 6, 7]. Over recent years, PVP has been used for sperm manipulation in human assisted reproduction (ART), and has been investigated during embryo development [8]. It is thus vital to confirm the safety of PVP application for human ART. Therefore, the objective of this review article is to examine the detrimental effects and potential risk of PVP upon sperm function and early embryo development following human ICSI.

## The history of PVP in assisted reproduction for animals

PVP has been used for sperm selection, oocyte culture and cryopreservation for the last 50 years. Initially, PVP, the average molecular weight of which is 10,000 Da, was found to provide the greatest degree of protection to platelets when cryopreserved at −196 °C [9]. Researchers demonstrated the production of piglets following the transfer of vitrified porcine embryos after stepwise dilution of PVP and other cryoprotectants [9]. Next, percoll, consisting of silica particles coated with PVP, have been used for many years for routine sperm preparation for animal reproduction protocols [10, 11, 12]. Motile sperm were highly purified in an inner column of a centrifuge tube via the use of a discontinuous percoll density gradient [10]. In another study, PVP was substituted for protein in media to promote the development of in vitro methods that permit IVM/IVF embryos to mature, fertilize and develop to the blastocyst stage, but in a protein‐free medium without bovine serum albumin [11, 12, 13]. PVP prevents oocytes from adhering to plastic and glass dishes. PVP was also used to establish a culture system to support the growth of immature bovine oocytes enclosed in granulosa cell complexes [14]. When PVP medium was used, the ability of immature oocytes obtained from bovine ovaries to fertilise and develop to the blastocyst stage was increased in vitro [14]. Finally, PVP has been used successfully for intracytoplasmic sperm injection (ICSI) in order to increase the viscosity of sperm solution, thus facilitating the handling and immobilization of individual sperm in both domestic animal and human situations [8, 15, 16, 17, 18, 19]. PVP can be used to help regulate fluid handling in the injection pipette and limit the final volume injected into the oocyte [20]. During ICSI, sperm are first suspended in a medium containing PVP and a single spermatozoon is chosen and injected into the oocyte, unavoidably together with a small amount of medium [21].

## Current perspective and potential risk for the application of PVP for intracytoplasmic sperm injection in clinics and hospitals

A summary of the clinical use of PVP is given in Table 1. More than 90 % of published studies used PVP to immobilize sperm motility during ICSI treatment. Fertilization, cleavage and clinical pregnancy rate were 20–80, 40–90 and 10–50 %, respectively. More than ten thousand ICSI children have been born every year since ICSI was introduced for human ART [22]. These data suggest that the embryo can develop to fetal stages and into a healthy child following the co‐injection of sperm and a small volume of PVP into the oocyte during infertility treatment.

**Table 1:** The use of PVP in clinical reports for 20 years

PVP used/not used	PVP brand	Fertilization rate^a^ (%)	Cleavage rate^b^ (%)	Clinical pregnancy rate^c^ (%)	References
×	–	80.0	80.0	0.0	[59]
o	–	41.7	41.7	50.0	[60]
×	–	68.0	52.9	14.3	[61]
×	–	69.0	13.3	44.6	[62]
o	–	66.7	75.0	50.0	[63]
o	–	86.7	15.4	16.7	[64]
o	–	73.9	22.4	40.9	[65]
o	–	–	–	–	[66]
o	–	–	–	–	[67]
o	–	–	–	–	[68]
o	–	–	–	–	[69]
×	–	–	–	–	[70]
×	–	–	–	–	[71]
o	–	–	–	–	[72]
o	–	82.6	69.5	–	[73]
o	ICN Biochemicals	–	–	–	[8]
o	Scandinavian IVF Science AB	64.3	–	28.4	[74]
o	Scandinavian IVF Science AB	67.6	62.2	17.0	[75]
o	SAGE IVF Inc.	66.7	54.4	46.8	[76]
o	VitroLife	58.3	95.7	37.0	[77]
o	VitroLife	73.0	–	31.3	[78]
o	VitroLife	71.3	92.8	40.9	[79]
o	Fertipro	59.3	55.8	46.3	[80]
o	Fertipro	57.0	54.0	18.1	[81]
o	Sigma	–	–	–	[82]
o	Sigma	19.0	14.2	50.0	[83]
o	Sigma	69.4	62.9	44.4	[84]
o	Sigma	–	25.0	25.0	[85]
o	Sigma	–	–	–	[86]
o	Sigma	80.9	88.3	26.7	[87]
o	MediCult	85.8	–	20.9	[88]
o	MediCult	56.3	–	37.1	[89]
o	MediCult	87.1	–	21.6	[90]
o	MediCult	80.9	78.9	40.6	[91]
o	MediCult	62.6	60.6	31.1	[92]
o	MediCult	67.0	51.3	28.9	[93]
o	MediCult	61.9	–	57.1	[94]
o	MediCult	27.0	27.0	33.3	[95]
o	MediCult	45.0	–	–	[96]
o	MediCult	75.6	75.6	44.1	[97]
o	MediCult	69.0	69.0	49.1	[98]
o	Irvine Scientific	58.6	84.1	–	[99]
o	Irvine Scientific	–	–	–	[100]
o	Irvine Scientific	40.0	40.0	33.3	[101]
o	Irvine Scientific	43.0	39.0	–	[102]
o	Irvine Scientific	73.9	82.7	28.6	[103]
o	Irvine Scientific	67.7	50.3	25.0	[104]
o	Irvine Scientific	67.9	–	37.5	[105]
o	Irvine Scientific	–	43.9	44.8	[106]
o	Irvine Scientific	–	–	–	[107]

^a^Fertilized oocytes per injected metaphase II oocyte

^b^Cleavaged oocytes per injected metaphase II oocyte

^c^The number of pregnancy per embryo transferred

However, the exposure of sperm to PVP has been found to cause sub‐microscopic changes in sperm structure; damage has been observed in the sperm nucleus, both in terms of shape and in the texture of chromatin, which was frequently decondensed [23]. PVP‐induced nuclear and membrane damage may have been due to the breakdown of sperm membranes [24]. These studies suggest that PVP induces nuclear damage in the sperm leading to subsequent chromosomal aberration. Furthermore, PVP delayed the onset of calcium oscillations and sperm decondensation within the oocyte [25, 26]. Consequently, it is likely that exposure of sperm to PVP may suppress fertilization and embryonic development. Moreover, as the molecular weight of PVP used for ICSI is 360,000 Da, PVP injected during the ICSI procedure remains in the oocytes for a prolonged period of time [27]. Consequently, this means that PVP is likely to impede embryo development and pregnancy.

The numbers of ICSI treatments have been increasing more than conventional IVF treatments in Europe over the last few years [28]. These data suggest that ICSI treatment for male fertility is becoming the most important option for human ART. However, the European pregnancy rates of ICSI embryos are lower compared to IVF embryos [28]. The main differences between ICSI and IVF treatments involve the oocyte membrane being broken by a micropipette, along with the subsequent injection of sperm, PVP solution and external media, or the process of fertilization, especially, non‐sperm‐egg fusion in ICSI, there is delay of onset of sperm decondensation and Ca oscillation [29].

## The detrimental effects of PVP upon sperm function

### PVP can cause injury to the sperm membrane, acrosome, head and nucleus

**Figure 1:**
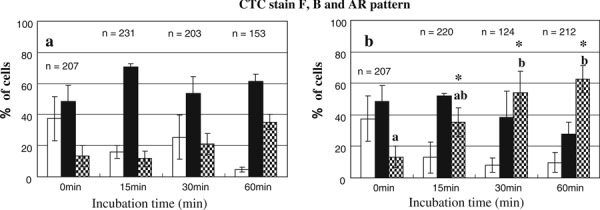
Effect of PVP on capacitation and the acrosome reaction in bovine sperm. Uncapacitated bovine sperm suspension was incubated for 0, 15, 30 and 60 min in the absence (**A**) or presence (**B**) of 10 % PVP in modified‐SOF and then analysed using CTC stain. Experiments were repeated six times. Data represent the percentage of cells expressing the F pattern (*open bars*), B pattern (*filled bars*), and AR pattern (*hatched bars*) of CTC fluorescence. Values represent mean ± SEM. Annotation (*a*, *b*) indicates *p* < 0.05 compared to 0 min (ANOVA: *F*_6,38_ = 0.0068). *Asterisks* indicate *p* < 0.05 compared to controls. *n* the number of sperm. Figure from Kato and Nagao [30]

We examined the effects of PVP upon sperm capacitation and the acrosome reaction in bovines [30] and found that sperm cultured in PVP demonstrated increased rates of acrosome reaction when compared with bull sperm cultured in a control group (Fig. 1). PVP may thus trigger the acrosome reaction. While numerous events are known to occur during capacitation, it appears that regulation of the intracellular concentration of Ca^2+^ is one of the most important. During capacitation, the initial influx of Ca^2+^ into the sperm is used to fill an intracellular Ca^2+^ store located in the acrosome [31]. One possibility suggested by Spungin and Breitbart [32] was that increased levels of adenylate cyclase activity triggered a further increase in cyclic AMP causing further Ca^2+^ channels in the acrosome to release Ca^2+^ from internal stores into the cytoplasm. Binding of capacitated sperm to the zona pellucida triggers the activation of a G protein that, in turn, opens a cation channel in the plasma membrane [33]. Furthermore, the acrosome reaction has been induced artificially following the influx of Ca^2+^ into the sperm head [34]. In human sperm, PVP has been shown to induce damage to sperm membranes, mitochondrial membranes and to cause deterioration of the axonemal tubules and fibrous sheath [23]. When considered collectively alongside these earlier studies, we infer that the presence of PVP in culture medium induces damage to the sperm plasma membrane and thus initiates the influx of Ca^2+^ into the sperm cells prematurely, thereby inducing the acrosome reaction.

### Sperm exposed to PVP incurred damage to the nucleus following ICSI

We also examined the effects of incubating bull sperm in PVP on fertilization [30] and found that sperm cultured in PVP solution exhibited lower fertility rates in vitro compared with sperm cultured in a control media. This finding showed that the use of PVP solution resulted in a significant reduction in the rate of sperm incorporation into oocytes. From this finding, we concluded that PVP caused injury to the ultrastructure of the mitochondria and sperm tail in our experiments. It is well established that sperm cells become motile and travel to oocytes via tail motion. The hyperactive state associated with the acrosome reaction allows sperm to penetrate the cumulus and zona pellucida surrounding the oocyte [35]. Progressive motility is one of the most important criteria for establishing the fertilization potential of sperm [36]. This finding demonstrates that incubation with PVP affects natural gamete interactions and can result in lower fertilization rates.

We also showed the effect of sperm cultured in PVP solution upon fertilization following ICSI. Most sperm cultured in PVP involved the acrosome reaction (Fig. 1), then the sperm were injected into bovine oocytes. These results showed that the injection of sperm without intact acrosomes and cultured in PVP, resulted in enhanced pronuclear formation following ICSI. The reason for this is that if the acrosome is removed from the sperm head, then the sperm nucleus may become pronuclear. Ca oscillations were immediately initiated after injection of acrosome‐less and demembranated mouse spermatozoa [29]. Generally, the factor influencing onset of Ca oscillation in ICSI most is immobilization of sperm [37]. PVP also delayed the onset of calcium oscillations in the oocyte [25, 26]. From these finding, PVP directly and indirectly influence Ca oscillation after sperm injection. Recent studies show that oscillatory patterns of Ca^2+^ in fertilized mouse eggs influence embryonic gene expression in blastocysts and post‐implantation development to term [38]. Data also suggest that hyper‐stimulation of the calcium oscillation profile exhibited a far greater variability in birth weight and pregnancy/implantation rates following embryo transfer. We infer that PVP‐treated sperm might affect the Ca^2+^ oscillation profile, resulting in impediment to embryo and fetus development. Therefore, PVP could be potentially dangerous for the sperm nucleus during ICSI treatments. Consequently, when embryologists use PVP solution, it may be better to reduce the length of the treatment period.

## PVP may induce defective embryo development

### The hazardous effects of PVP injection upon pronuclear oocytes and development to the blastocyst stage

Embryo development was clearly suppressed by PVP injection in bovines [30]. Rates of cleavage and blastocyst formation were significantly reduced in embryos injected with PVP (Table 2). Embryos injected with PVP mostly arrested at the two‐ to 16‐cell stage (data not shown) and only a few developed to the blastocyst stage. The total numbers of cells at the blastocyst stage in control groups were higher than in the PVP group (88.9 ± 6.0, 90.4 ± 4.5, and 48.8 ± 10.7, respectively). There was no difference in the chromosomal integrity of blastocysts when compared between PVP‐injected blastocysts and non‐injected blastocysts (64.3 %:9/14 vs 78.9 %:15/19, *p* > 0.05). In this particular experiment, the direct injection of PVP into IVF embryos resulted in a reduction in the rate of cleavage and blastocyst formation, along with a reduction in the number of cells in blastocysts when compared with control IVF embryos (Table 2). In the first cleavage, microfilaments play a key role in mitosis and cytokinesis [39], indicating that PVP‐induced deterioration of the axonemal tubules [23], and thus microfilaments, could impart significant detriment upon in vitro development of the embryo, cleavage and blastocyst stage. On the other hand, PVP did not affect chromosomal integrity in this particular study, although other authors [40] have suggested that PVP may affect chromosomes for long periods throughout the blastocyst stage. Moreover, the effects of maintaining mouse sperm in PVP solution upon developmental arrest [41], and the effect of injecting mouse zygotes with small amounts of medium, may serve as key research for human ICSI, while avoiding ethical problems linked with experiments with human oocytes and embryos [42].

**Table 2:** In vitro developmental rate of bovine embryos to the blastocyst stage following intracytoplasmic injection with PVP solution (*n* = 3–9)

	Number of oocytes	Number of embryos cleaved (%)	Number of blastocysts (%)
Injected product			
None (control)	129	105 (81.4 %)^a^	31 (24.0 %)^c^
SOF	79	62 (78.5 %)^a^	19 (24.1 %)^c^
PVP	73	38 (52.1 %)^b^	5 (6.8 %)^d^
Brand of PVP injected			
Sigma	90	44 (48.9 %)^e^	13 (14.4 %)^gh^
Irvine	115	79 (68.7 %)^e^	24 (20.9 %)^g^
Fertipro	110	53 (48.2 %)^f^	9 (8.2 %)^h^
Injection medium			
199	65	41 (63.1 %)	9 (13.8 %)
SOF	95	63 (66.3 %)	11 (11.5 %)
HTF	61	36 (59.0 %)	6 (9.8 %)

This table was from Kato and Nagao [30]

*n* number of replicated experiments, letters within columns indicate significant differences (^a–b^ *χ* ^2^ = 10.142, *p* < 0.001, ^c–d^ *χ* ^2^ = 10.14, *p* < 0.01, ^e–f^ *χ* ^2^ = 12.09, *p* < 0.01, ^g–h^ *χ* ^2^ = 7.260, *p* < 0.05)

### Media brand could impede early embryo development while solvents of PVP do not

The differing nature of the various brands of PVP is known to cause differential effects. We infer that the process used to manufacture PVP can cause influence upon embryo development due to differences among various brands and solvent media [30]. The level of PVP purification, and therefore, the potential for contamination, may be critical in the generation of more efficient techniques for performing human ICSI. Van Steirteghem et al. [8] used dialyzed PVP for human ICSI. Thus, there remains the distinct possibility that the level of purification and potential contamination associated with PVP brands A, B and C may exert differential effects upon embryonic development. It is therefore very important for embryo development that different chemical suppliers are investigated in this respect [43]. Clearly, it would be vital to avoid selecting any media that was known to suppress embryo development.

We also investigated the brand of PVP in many clinical reports (Tables 1, 3). The average of fertilization rates were 58.5 % (Irvine), 58.2 % (Fertipro), 56.4 % (Sigma), 67.5 % (Vitrolife) and 65.8 % (Medicalt), respectively (Tables 1, 3 show reference). The average of cleavage rates were 56.7 % (Irvine), 54.9 % (Fertipro), 47.6 % (Sigma), 94.3 % (Vitrolife) and 60.4 % (Medicalt), respectively. The average of clinical pregnancy rates were 33.8 % (Irvine), 32.2 % (Fertipro), 36.5 % (Sigma), 36.4 % (Vitrolife) and 36.4 % (Medicalt), respectively (Tables 1, 3 show reference). There were no differences between the pregnancy rates for Irvine, Fertipro, Sigma, Vitrolife and Medicalt media. From these studies, differences were apparent between different brands in terms of embryo development and development to fetus, at least in the bovine model. However, in clinical reports, these rates were not significantly affected by the solvent used to dilute the PVP (Table 3).

**Table 3:** PVP availability reported during the past 20 years

Use/or not PVP	PVP brand	Fertilization rate^a^ (%)	Cleavage rate^b^ (%)	Pregnancy rate^c^ (%)
○	Scandinavian IVF Science AB (*n* = 2)	66.0	62.2	22.7
○	SAGE IVF Inc. (*n* = 1)	66.7	54.4	46.8
○	VitroLife, Kungsbacka, Sweden (*n* = 3)	67.5	94.3	36.4
○	Fertipro, Belgium (*n* = 2)	58.2	54.9	32.2
○	Sigma, St. Louis, MO, USA (*n* = 3–4)	56.4	47.6	36.5
○	MediCult, Jyllinge, Denmark (*n* = 7–12)	65.3	60.4	36.4
○	Irvine Scientific, Santa Ana, Ca, USA (*n* = 5–6)	58.5	56.7	33.8

^a^The average of fertilization rates in the reports (*n*)

^b^The average of cleavage rates in the reports (*n*)

^c^The average of pregnancy rates in the reports (*n*)

Other factors to consider when addressing the potential utility of PVP are those pertaining to the PVP solvent selected for use. We investigated PVP solvents in several clinical reports. The composition of PVP solvent media are shown in Table 4. There were no differences between the pregnancy rates when different types of solvent were compared (Tables 1, 3, 4). From these studies, it appears that the solvent used for PVP does not affect embryo development and development of the fetus.

**Table 4:** Composition of injection media for human intracytoplasmic injection of PVP

Fertipro	MediCult	SAGE IVF Inc (present; CooperSurgical, Inc.)	Irvine Scientific	VitroLife
Water	Water	Water	Water	Water
Sodium chloride	–	Sodium chloride	Sodium chloride	Sodium chloride
Potassium chloride	–	Potassium chloride	Potassium chloride	Potassium chloride
Calcium chloride	–	Calcium chloride	Calcium chloride, anhydrous	Calcium chloride
Sodium dihydrogen phosphate	–	–	–	–
–	–	Potassium phosphate, anhydrous	Potassium phosphate, monobasic	Potassium di‐hydrogen
Magnesium sulfate	–	Magnesium sulfate	Magnesium sulfate, anhydrous	Magnesium sulfate
Sodium pyruvate	Sodium pyruvate	Sodium pyruvate	Sodium pyruvate	Sodium pyruvate
Glucose monohydrate	Glucose	Glucose	Glucose	Glucose
Sodium lactate	–	Sodium lactate	Sodium lactate	Sodium lactate
Sodium bicarbonate	Sodium bicarbonate	Sodium bicarbonate	Sodium bicarbonate	Sodium bicarbonate
		Taurine		
		Alanyl‐glutamine		
HEPES	Hepes free acid	HEPES	–	–
–	Hepes sodium salt	–	–	–
–	–	EDTA	–	EDTA
Human serum albumin	Human serum albumin	Human serum albumin 5 mg/mL	Human serum albumin 7–10 %	Recombinant human albumin
Polyvinylpyrrolidone	Polyvinylpyrrolidone	Polyvinylpyrrolidone	Polyvinylpyrrolidone	Polyvinylpyrrolidone
–	Streptomycine sulfate salt	Gentamicin	Gentamicin	–
–	Penicillin sodium salt	–	–	–
–	Phenol red (not product no. 1090)	Phenol red	–	–
–	EBSS		–	–
	Synthetic Serum Replacement (SSR^®^; USA: ART supplement contains recombinant human insulin)	–	–	–

### PVP can localize inside oocyte/embryo and prevent development to the fetal stage

During ICSI, PVP molecules are injected directly into the oocytes and their effect on intracellular membranes or DNA in the developing embryo is unknown. No detrimental effects have been demonstrated in the development of preimplantation bovine embryos in vitro [44]. However, our results showed that the direct injection of PVP into IVF embryos resulted in a reduction in the rates of cleavage and blastocyst formation, along with a reduction in the number of cells in blastocysts, relative to control IVF embryos. The reason for this was the smaller volume used by Motoishi et al. [44] compared to that used by Kato et al. [29]. Motoishi et al. injected only 2–3 pl of PVP into the bovine zygote while Kato et al. injected 24–32 pl of PVP. We conclude that the larger volume reduced development to the blastocyst stage, along with blastocyst cell number.

**Figure 2:**
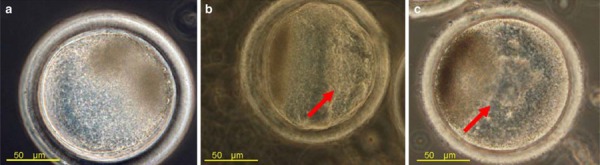
The location of PVP solution in embryos. **a** PVP was generally not observed in the embryo and appeared similar to the control. **b** PVP generally dispersed with some PVP remaining at the injection site. **c** Entire volume of PVP remaining in parts of the oocyte. *Arrows* show the localization of PVP solution. Figure from Kato and Nagao [30]

We demonstrated the precise localization of PVP solution in embryos. An example of a fixed IVF embryo is shown in Fig. 2. There were three patterns of PVP location in fixed IVF embryos. In most embryos, PVP solution dispersed soon after injection (1–3 h) and was not evident in the IVF embryos; similar results were obtained in the control group (Fig. 2a). In some IVF embryos, PVP generally dispersed (59.1 %), although some still remained at the injection site (15.9 %, Fig. 2b). In other embryos, all of the PVP solution remained at the injection site (25.0 %, Fig. 2c). PVP remained in 40.9 % of PVP‐injected IVF embryos.

Since PVP is a large polymer (molecular weight 360 kDa), it will therefore be difficult to diffuse out of the oocyte or be readily digested by lysosomal enzymes [27]. Cells that are routinely in contact with PVP exhibited increased mucoid secretion as a result of the interaction between this agent and the cell cytoplasm [45]. PVP is likely, therefore, to be present in the inner cell mass and trophectoderm, and be transferred to organs and tissue of the fetus and induce cell death as a result of organelle damage. Previously, our studies showed that PVP injection reduced the cell number of blastocysts [30]. The number of trophectoderm cells is related to pregnancy and implantation in human ART [46]. In clinical reports describing ICSI with or without PVP, the mean averages of clinical pregnancy rates were 47.4 % [47, 48, 49] and 33.1 % (Table 1), respectively. Moreover, the application of PVP conveys potential embryonic toxicity which may result in chromosomal abnormalities [27]. It has been reported that chromosomal abnormalities in ICSI‐derived pregnancies might be related to the inclusion of PVP during the ICSI procedure [39]. These data imply a hazardous risk that PVP impedes blastocyst quality and pregnancy, and might induce the miscarriage of ICSI embryos. On the other hand, spontaneous abortion among 29–39 years olds was approximately 10–25 % [50], and the rate amongst IVF and ICSI cases was 11.5–12.3 and 10.6–12.3 % in the UK between 2002 and 2005, respectively [51, 52, 53, 54]. There were no differences in abortion rates among natural, IVF and ICSI cases. Therefore, we concluded that while spontaneous abortion is not induced by human ART, it is possible that PVP retained within ICSI embryos may induce miscarriage during pregnancy.

## The use of immobilization media without PVP for ICSI in clinical treatment

HA is an anionic, nonsulfated glycosaminoglycan distributed widely throughout connective, epithelial, and neural tissues. Hyaluronan contributes significantly to cell proliferation and migration in the extracellular matrix [55]. Polymers of hyaluronan range in size from 5,000 to 20,000,000 Da in vivo. [56]. Hyaluronate is degraded to natural sugar molecules that can be metabolized readily by normal cellular biochemical pathways by lysosomes [57]. Moreover, the selection of normal sperm by hyaluronic acid binding assays might help to reduce early embryonic mortality due to chromosomal aberration [58]. We reviewed the effect of HA and PVP as sperm handling solutions during ICSI treatments (Table 5). There were no differences between the fertilization and pregnancy rate following PVP‐ICSI and HA‐ICSI. These data suggested that hyaluronate could become a direct replacement for PVP, as a natural and readily degradable glycosaminoglycan [58].

**Table 5:** The risk of PVP and HA during ICSI treatments

	Report A [109]		Report B [108]		Report C [88]		Report D [90]	
	Group HA	Group PVP	Group HA	Group PVP	Group HA	Group PVP	Group HA	Group PVP
Number of cycles	58	65	48	44	125	107		
Fertilization rate^a^	72.6 % (525/723)	74.6 % (484/649)	72 % (360/499)	75 % (337/449)	91.6 % (304/332)	85.8 % (236/275)	93.4 % (874/936)	87.1 % (223/256)
Clinical pregnancy rate^b^	50 % (29/58)	38.5 % (25/65)	41.7 % (20/48 )	43.2 % (19/44)	24.8 % (31/125)	20.9 % (22/105)	32.8 % (107/326)	21.6 % (21/96)
Implantation rate^c^	18.6 % (41/221)	14.0 % (35/250)	18.1 % (27/149)	19.1 % (27/141)	12.4 % (35/282)	10.2 % (23/226)	17.1 % (133/778)	10.3 % (22/213)

^a^Fertilized oocytes per injected metaphase II cumulus–oocytecomplex

^b^The number of pregnancy per embryo transferred

^c^Tnumber of gestation sacs per embryo transferred

Sperm immobilization medium that was devoid of PVP has been used to perform ICSI in Kato Ladies’ Clinic and their associated clinical groups. Fertilization and blastocyst rates were more than 80 and 50 %, and clinical pregnancy rates exceeded 40 % [47, 48, 49]. These clinics have helped many infertile couples and resulted in the birth of more 20,000 children. PVP‐free solution would require the high level technique of sperm immobilization and manipulation and improve the technique of human embryologist. These technical improvements could increase the embryo quality and pregnancy rate. The embryologists should select a lower concentration of PVP solution for ICSI treatment, and undergo effective training in order to perform future ICSI cycles without PVP. For direct and indirect reasons, the success rate of fertilization and clinical pregnancy in human ICSI may be improved by using PVP‐free solution during ICSI.

## Conclusion

PVP can cause significant damage to sperm membranes and induce the acrosome reaction and reduce fertilization rate. Moreover, PVP remained detectable in IVF embryos, suppressed embryo development, and reduced the number of cells at the blastocyst stage. Clinical pregnancy rates of ICSI using sperm immobilization without PVP exhibited high rates. Miscarriage might be related to the injection of sperm with PVP during human ICSI. On the other hand, the immobilization of sperm in PVP‐free media for ICSI is difficult for junior or trainee embryologists. Sperm immobilization in PVP‐free media could be beneficial for the technical improvements, embryo quality and pregnancy rate in the hands of an experienced embryologist.

## Acknowledgments

The authors thank Genetics Hokkaido Co., Ltd for providing frozen semen and Chikusei Meat Center and Nakao Chikusan Co., Ltd. for providing the ovaries used in this study.

### Open Access

This article is distributed under the terms of the http://creativecommons.org/licenses/by/3.0/ License which permits any use, distribution, and reproduction in any medium, provided the original author(s) and the source are credited.
